# New Books

**Published:** 2007-12

Accounting for Climate Change: Uncertainty in Greenhouse Gas Inventories—Verification, Compliance, and Trading

D. Lieberman, M. Jonas, Z. Nahorski, S. Nilsson, eds.

New York:Springer, 2007. 162 pp. ISBN: 978-1-4020-5929-2, $139

American Environmental Policy, 1990–2006: Beyond Gridlock

Christopher McGrory Klyza, David Sousa

Cambridge, MA:MIT Press, 2007. 408 pp. ISBN: 978-0-262-11313-7, $69

Climate and Land Degradation

Mannava V.K. Sivakumar, Ndegwa Ndiang’ui, eds.

New York:Springer, 2007. 623 pp. ISBN: 978-3-540-72437-7, $229

Earth Science and Applications from Space: National Imperatives for the Next Decade and Beyond

Committee on Earth Science and Applications from Space, National Research Council

Washington, DC:National Academies Press, 2007. 456 pp. ISBN: 978-0-309-10387-9, $46

Environmental Modeling

Ekkehard Holzbecher

New York:Springer, 2007. 392 pp. ISBN: 978-3-540-72936-5, $139

Health Effects of Beryllium Exposure: A Literature Review

Committee on Beryllium Alloy Exposures, Committee on Toxicology, National Research Council

Washington, DC:National Academies Press, 2007, 118 pp. ISBN: 978-0-309-11167-6, $26.32

Highway and Urban Environment

Gregory M. Morrison, Sébastien Rauch, eds.

New York:Springer, 2007. 594 pp. ISBN: 978-1-4020-6009-0, $239

Implementing Integrated Water Resources Management in Central Asia

Patricia Wouters, Victor Dukhovny, Andrew Allan, eds.

New York:Springer, 2007. 177 pp. ISBN: 978-1-4020-5730-4, $159

Isotopes in the Water Cycle: Past, Present and Future of a Developing Science

Pradeep K. Aggarwal, Joel R. Gat, Klaus F. Froehlich, eds.

New York:Springer, 2007. 381 pp. ISBN: 978-1-4020-6671-9, $59.95

Particle-Laden Flow

Bernard J. Geurts, Herman Clercx, Wim Uijttewaal, eds.

New York:Springer, 2007. 426 pp. ISBN: 978-1-4020-6217-9, $169

Review of the U.S. Climate Change Science Program’s Synthesis and Assessment Product 3.2, “Climate Projections Based on Emission Scenarios for Long-lived and Short-lived Radiatively Active Gases and Aerosols”

Committee to Review the U.S. Climate Change Science Program’s Synthesis and Assessment Product 3.2, National Research Council

Washington, DC:National Academies Press, 2007. 54 pp., free downloads

The Environment in Asia Pacific Harbours

Eric Wolanski, ed.

New York:Springer, 2007. 498 pp. ISBN: 978-1-4020-6566-8, $99

The Global Circulation of the Atmosphere

Tapio Schneider, Adam H. Sobel, eds.

Princeton, NJ:Princeton University Press, 2007. 400 pp. ISBN: 978-0-691-12181-9, $65

The Impact of The WTO: The Environment, Public Health and Sovereignty

Trish Kelly

Northampton, MA:Edward Elgar, 2007. 232 pp. ISBN: 978-1-84720-081-5, $90

Water Implications of Biofuels Production in the United States

Committee on Water Implications of Biofuels Production in the United States, National Research Council

Washington, DC:National Academies Press, 2007. 86 pp. ISBN: 978-0-309-11361-8, $18

